# An RNA Aptamer Targets the PDZ-Binding Motif of the HPV16 E6 Oncoprotein

**DOI:** 10.3390/cancers6031553

**Published:** 2014-07-24

**Authors:** Tamara A. Belyaeva, Clare Nicol, Özlem Cesur, Gilles Travé, George Eric Blair, Nicola J. Stonehouse

**Affiliations:** 1 School of Molecular and Cellular Biology, Faculty of Biological Sciences and Astbury Centre for Structural Molecular Biology, University of Leeds, Leeds LS2 9JT, UK; E-Mails: belyaeta@hotmail.com (T.A.B.); c.nicol@leeds.ac.uk (C.N.); bs09oc@leeds.ac.uk (Ö.C.); g.e.blair@leeds.ac.uk (G.E.B.); 2 UMR 7242 CNRS-Université de Strasbourg, Ecole Supérieure de Biotechnologie, Boulevard Sébastien Brant, Illkirch 67412, France; E-Mail: gilles.trave@unistra.fr

**Keywords:** HPV16 E6, PDZ domain, p53, RNA aptamer, SELEX

## Abstract

Human papillomavirus 16 (HPV16) is a high-risk DNA tumour virus which is the primary causative agent of cervical cancer. Cell transformation arises from deregulated expression of the E6 and E7 oncogenes. E6 has been shown to bind a number of cellular proteins, including p53 and proteins containing a PDZ domain. This study reports the first RNA aptamers to E6. These have been employed as molecular tools to further investigate E6-p53 and E6-PDZ interactions. This study is focussed on two aptamers (termed F2 and F4) which induced apoptosis in cells derived from an HPV16-transformed cervical carcinoma. The molecules were able to inhibit the interaction between E6 and PDZ1 from Magi1, with F2 being the most effective inhibitor. Neither of the aptamers inhibited E6-p53 interaction or p53 degradation. This study shows the specificity of this approach and highlights the potential benefits of the E6 aptamers as potential therapeutic or diagnostic agents in the future.

## 1. Introduction

Human papillomaviruses (HPVs) are DNA tumour viruses that infect epithelial cells. More than 100 types have been identified and those which infect genital epithelia are classified as low- or high-risk, depending on the risk of development of cancer [1,2]. High-risk viruses cause a range of anogenital and oropharyngeal tumours, most commonly cervical cancer, and have also been associated with squamous cell carcinoma of the head and neck and with Bowen’s disease [3,4]. Of the high-risk types, HPV16 is the most common causative agent of cervical cancer [5]. Cellular transformation involves the viral oncoproteins E6 and E7 [6,7]. E6 has been shown to promote degradation of the tumour suppressor p53 by interacting with the E3 ubiquitin ligase, E6-AP [8,9] while E7 has been demonstrated to bind and destabilise the cell cycle control protein pRb [10,11]. In addition to these well characterised roles, E6 and E7 interact with at least 50 other cellular proteins (for reviews see [12,13]). One of the key interactions is E6 targeting of proteins containing a PDZ domain (post synaptic density protein, *Drosophila* disc large tumour suppressor, zonula occludens-1 protein). However, the role of this interaction in cellular transformation is not fully understood. PDZ-domain containing proteins include hScrib [14], MUPP1 [15] and members of the membrane-associated guanylate kinase MAGUK family such as Magi1 and Dlg [16,17,18,19]. E6 binds to these multi-domain proteins through a short C-terminal PDZ-domain-binding motif (ETQV) [16] which sometimes results in proteasome-induced degradation of the targeted PDZ-proteins. E6 is also able to bind to DNA, recognising four-way Holliday junctions [20] and to RNA [21], possibly playing role in RNA splicing [22]. HPV16 E6 is a protein of approximately 150 amino acids, possibly functional as a homodimer, with zinc-binding domains at both N- and C-termini [23]. The solution structure of the C-terminal domain of HPV16 E6 [21] has revealed a novel zinc-binding fold with a positively-charged surface favouring the interaction with nucleic acids.

While HPV vaccination may be a significant development in prevention of HPV-associated cancer, the current vaccines are designed to target only a subset of high-risk HPV types (HPV16/18) and several other high-risk HPVs exist. Furthermore, due to the prolonged latency period, the time-scale for clinical benefit is likely to be long. A better understanding of the disease process, together with novel therapeutic approaches is therefore required. Aptamers are single-stranded oligonucleotides, produced by the iterative process termed systematic evolution of ligands by exponential enrichment or SELEX [24,25,26], that fold into complex structures and bind target molecules in a conformation-dependent manner. Aptamers can be stabilised against degradation and can be modified to render them non-immunogenic (for reviews see [27,28,29]). Furthermore, because of the high affinity of aptamer binding, these molecules have the ability to modulate the function of target molecules and therefore have therapeutic potential. Examples of this technology include the aptamer Macugen^®^ (also known as pegaptanib) which gained clinical approval in 2004 to treat age-related macular degeneration [30], and G-rich DNA oligonucleotides (e.g., AS1411) with anti-proliferative properties in cancer cells [31,32].

We have previously reported the selection and characterisation of RNA aptamers to E7 [33,34].One of these molecules (termed A2) was able to induce apoptosis in SiHa cervical carcinoma cells (which express HPV16 E6 and E7) and target E7 for degradation. Here, we describe the selection of aptamers to E6. We have focussed on two molecules that were able to inhibit the interaction between E6 and PDZ1 of Magi1 and induce apoptosis in SiHa cells. In contrast, the aptamers had little effect on the E6-p53 interaction. This demonstrates the specificity of this approach and the potential for these molecules as novel therapeutics in the future.

## 2. Results and Discussion

### 2.1. Selection of RNA Aptamers to E6

**Figure 1:**
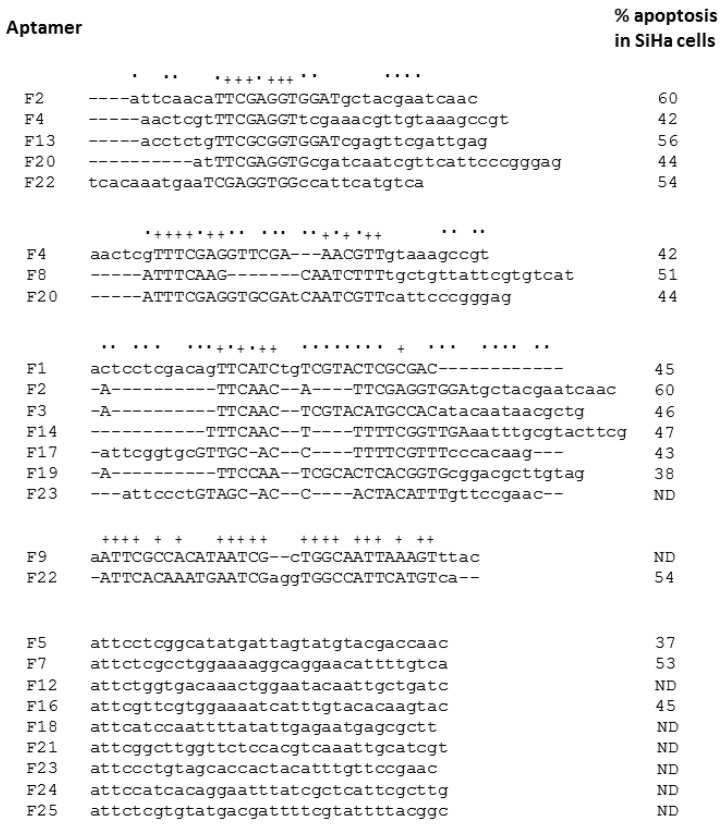
Individual aptamer sequences. Following 13 rounds of SELEX, RNA selected for binding to GST-E6 was reverse transcribed into cDNA and amplified by PCR. The resulting DNA template pool was cloned into the pGEM-T Easy vector and individual clones were isolated and sequenced. Sequence alignment of the random regions of the molecules was performed using the GeneBee multiple sequence alignment tool (AliBee). Capitals denote sequence motifs, identical sequences are indicated by +, identical in some alignments by ˙. Also shown is the % of apoptosis in SiHa cells induced by 50 nM of selected aptamers (*n* = 1). ND = not determined.

Thirteen rounds of *in vitro* selection were performed with GST-E6 as the target protein, immobilised on glutathione-sepharose beads (GS-beads) and an RNA pool modified with 2'F pyrimidines to increase RNA stability. Negative selection against beads bound to GST alone was included prior to each round of positive selection against GST-E6-bound beads. From the round 13 pool, individual molecules were cloned and sequenced. Analysis of 22 sequences by multiple alignment revealed some sequence identity between the aptamers (Figure 1). Aptamers F9 and F22 were the most similar (over 60%) while F2, F4, F13, F20 and F22 shared a common motif and 17% identity in the variable N_30_ regions. Overall, there was less sequence identity between the aptamer sequences than previously observed with aptamers selected to GST-E7 [33].

In order to determine which of the aptamers to take forward for study, a panel of molecules were screened for the ability to induce apoptosis in cells expressing E6. The SiHa cell line was used as this is derived from a human cervical carcinoma, has one or two copies of the HPV16 genome integrated into chromosome 13 and constitutively expresses both E6 and E7. SiHa cells were transfected with 50 nM of aptamer complexed to Oligofectamine. After 24 h, samples were dual-stained with FITC-conjugated annexin V and propidium iodide to identify apoptotic cells by flow cytometry. An increase in apoptosis was detected in cells transfected with all of the aptamers tested (ranging from 37% to 60%). Based on these data (Figure 1), aptamers F2 and F4 were selected for further study. F2 was included as one of the most pro-apoptotic aptamers tested (with 60% apoptosis) and F4 was chosen because of its similarity to F2 (the sequences of both aptamers include a TTCGAGGT motif) but showing a reduced apoptotic effect (42%).

**Figure 2:**
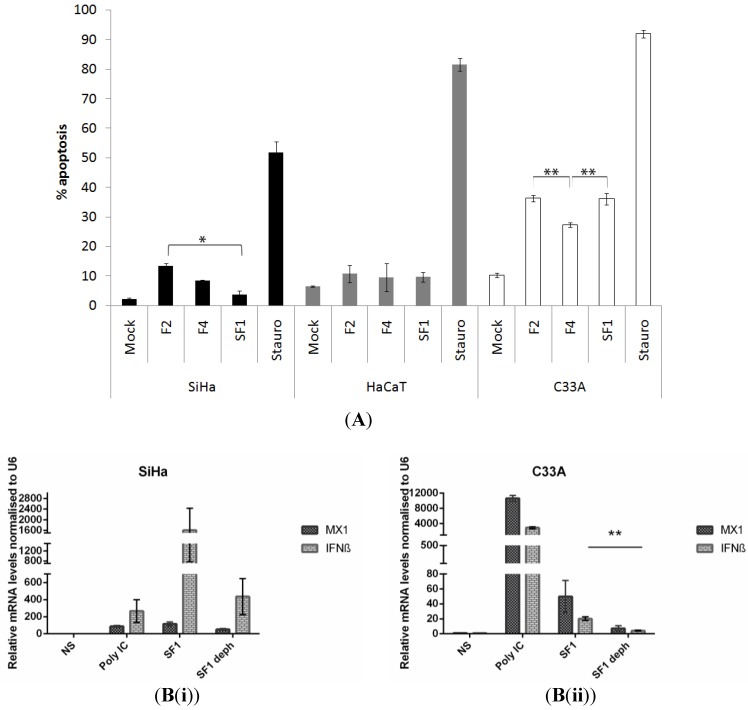
(**A**) Induction of apoptosis by aptamer treatment of HPV16- and non-virally-transformed human cells. SiHa, HaCaT and C33A cells were mock-treated or transfected with F2, F4 or control aptamer SF1 at a final concentration of 20 nM and analysed for apoptosis after 24 hours. Cells were dual-stained with FITC-conjugated annexin V and propidium iodide and analysed by flow cytometry. Graphs show total % apoptosis. Staurosporine treatment is included as a control. Results of three independent experiments and standard errors are shown. For clarity, p values between aptamers F2, F4 and SF1 only are shown (* = *p* < 0.05, ** = *p* < 0.01). (**B**) Effects of the 5'-de-phosphorylation of the RNA on the expression of interferon-response genes, MX1 and IFNβ. SiHa (**i**) and C33A (**ii**) cells were transfected with 100 nM SF1 ± de-phosphorylation (deph). After 24 h incubation, RNA levels were analysed by quantitative real-time PCR (qPCR). The level of gene expression in SF1, dephosphorylated SF1 and poly IC control treated samples was compared with that of the mock-transfected samples after normalisation to the expression level of the housekeeping gene, U6. Data from three independent experiments is shown, error bars show standard errors (** = *p* < 0.01).

To further investigate the effects of F2 and F4 on apoptosis, assays were performed (in SiHa cells together with HaCaT and C33A cells) at lower aptamer concentrations, in order to minimise non-specific effects. HaCaT cells are immortalised keratinocytes and C33A cells are derived from an HPV-negative cervical carcinoma. Neither express E6 or E7. Cells were transfected with 20 nM aptamer. An aptamer selected to an unrelated protein (RNA-dependent RNA polymerase of foot-and-mouth disease virus, FMDV), termed SF1 was included to control against non-specific effects of transfection with chemically modified RNA, as documented previously [34]. After 24 h, samples were dual-stained with FITC-conjugated annexin V and propidium iodide to identify apoptotic cells by flow cytometry. Data are shown, comparing the different levels of apoptosis induced by the three aptamers and staurosporine treatment. As illustrated in Figure 2, an increase in apoptosis was observed in SiHa cells transfected with 20 nM of either F2 or F4 (13.3% ± 1.0% and 8.5% ± 0.2%) respectively, over mock-transfected cells (2.2% ± 0.5%). The effect of F2 (but not F4) was significantly enhanced over that of SF1 (3.8% ± 1.3%, *p* = 0.024). In HaCaT cells, the corresponding levels of apoptosis were 10.8% ± 2.9% and 9.5% ± 4.8% for F2 and F4 respectively. Although these values are higher than the level of apoptosis measured in mock-transfected cells (6.5% ± 0.2%), they are similar to the levels measured after transfection with the control aptamer SF1 (9.7% ± 1.7%). These are likely to be non-specific effects as there were no statistically significant differences between treatment with F2, F4 or SF1. As a further control, the analysis was performed using the C33A cell line. These cells were highly susceptible to apoptosis. Transfection of all aptamers resulted in increased levels of apoptosis, compared to mock-treatment. However, in common with non-virally-transformed HaCaT cells, there were no significant increases in the levels of apoptosis induced by F2 or F4 (36.3% ± 1.0% and 27.3 ± 0.8%, respectively) in comparison with the control aptamer, SF1 (39.1% ± 1.9%). In fact, treatment with F4 resulted in a significant reduction in the level of apoptosis, in comparison to both SF1 and F2 (*p* = 0.008 and *p* = 0.001, respectively).

In order to investigate the non-specific effects of aptamer RNA on apoptosis, qRT-PCR experiments were performed, measuring the level of two interferon responsive genes (MX1 and IFNβ) in both SiHa and C33A cells. All of the aptamers described here are produced by *in vitro* transcription and will therefore incorporate a 5'-triphosphate. Although the aptamers are predicted not to contain extensive ds regions, the 5'-triphosphate can also be an innate immune trigger [35]. The effect of aptamer dephosphorylation on both MX1 and IFNβ was therefore evaluated, using poly IC as a control, Figure 2B. Dephosphorylation of SF1 appeared to result in a reduced response, however, this was only significant for IFNβ in C33A cells. This does provide support for the argument that at least some of the apoptotic effects seen were due to 5' end sensing. However, despite the different genetic background of the cells used here and possible differences in transfection efficiency, taken together, the data presented in Figure 2A suggest that aptamer F2 targets E6 in SiHa cells, thus inducing apoptosis.

### 2.2. Interaction of Aptamers with E6

**Figure 3:**
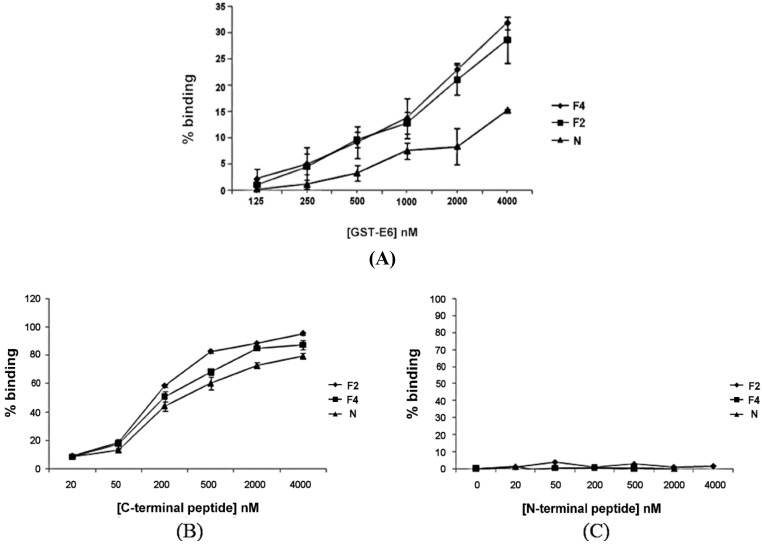
Binding of aptamers F2 and F4 and the naïve pool (N) to E6. (**A**); GST-E6-immobilized GS-beads were incubated with [^32^P]-labelled aptamers for 30 min at room temperature. After incubation, beads were washed and bound and unbound fractions quantified by scintillation counting. Magnetic beads coupled to synthetic peptides corresponding to either the (**B**) C-terminal or (**C**) N-terminal extremities of E6 were incubated with [^32^P]-labelled aptamers for 30 min at room temperature. After incubation, beads were washed and unbound fractions were quantified by scintillation counting. Data (presented as percentage bound) are from three separate experiments, standard errors are shown.

To assess the binding of F2 or F4 and GST-E6 in comparison to the naïve RNA pool, *in vitro* binding assays were performed (Figure 3A). [^32^P]-labelled aptamer, at a concentration of 1–2 nM, was bound to increasing concentrations of GST-E6 immobilised on glutathione-sepharose. Bound RNA was quantified by scintillation counting and expressed as a percentage of the total (*i.e.*, bound + unbound fractions). Because of the binding capacity of the GS-beads, binding of labelled aptamer did not reach saturation. Therefore it was not possible to estimate binding affinities, however it is clear that both F2 and F4 bound to GST-E6 at higher levels than the naïve RNA pool (Figure 3A). A similar, relatively modest increase in binding was seen previously during selection of aptamers to a nucleic-acid binding protein, the RNA-dependent RNA polymerase of FMDV. Despite this, some of the aptamers exhibited IC_50_ values of approximately 10–20 nM [36]. 

The C-terminal region of E6 has been previously shown to bind to proteins that contain PDZ-domains and also to E6AP, whereas the E6-p53 interaction involves both the N- and C-terminal regions of E6 [37,38]. In order to start to define the site of aptamer binding, further assays were performed using synthetic peptides corresponding to either the N- or C-terminal extremities of E6. Both F2 and F4 bound to the peptide derived from the C-terminus (Figure 3B), as did the naïve RNA pool, to a similar extent, but no binding to the N-terminal peptide was detected (Figure 3C). This could indicate that aptamers F2 and F4 were preferentially binding to the C-terminal region of E6. However, it should be noted that the C-terminal peptide was very positively charged and therefore the results could be due to a non-specific charge interaction. In order to probe this further, the effects of the aptamers on the interaction between E6 and PDZ-proteins were investigated.

### 2.3. Effect of F2 and F4 on E6-PDZ Interactions

**Figure 4:**
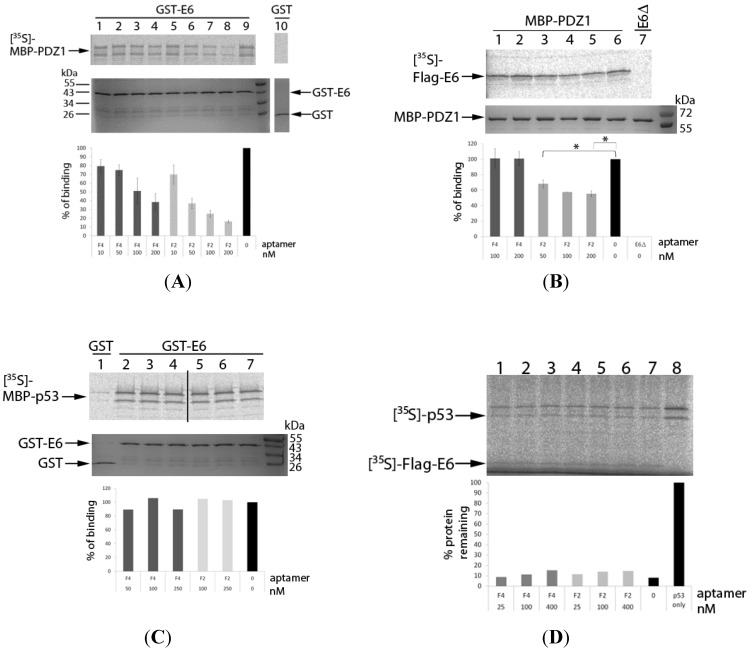
Effects of aptamers on the interactions of HPV16 E6 with some of its cellular binding partners. (**A**) Binding of MBP-PDZ1 to GST-E6. GST-E6-immobilized GS-beads were pre-incubated in the presence (lanes 1–8) or absence (lane 9) of F2 or F4 prior to addition of [^35^S]-MBP-PDZ1 and incubation for 1 h. GST-immobilized GS-beads were used as a control (lane 10). After incubation, beads were washed and proteins resolved by SDS-PAGE and analysed by autoradiography. A second gel was Coomassie-stained as a loading control for GST-E6. Binding (as a percentage of the no aptamer control) is shown graphically below the gel. It should be noted that MBP-PDZ-1 appears as two bands. Data from two independent experiments are shown, together with standard errors; (**B**) Binding of FLAG-E6 to MBP-PDZ1. [^35^S]-FLAG-E6 was pre-incubated in the presence (lanes 1–5) or absence (lane 6) of F2 or F4 at 4 °C. MBP-PDZ1-amylose resin was added and incubation continued for another hour. [^35^S]-FLAG-E6Δ, which lacks the C-terminal PDZ-binding domain, was used as a control for specificity of binding, in the absence of aptamer (lane 7). After incubation, the beads were washed and proteins resolved by SDS-PAGE and autoradiography. A second gel was Coomassie-stained as a loading control for MBP-PDZ1. Binding (as a percentage of the no aptamer control) is shown graphically below the gel. Data from three independent experiments are shown, together with standard errors, * = *p* < 0.05, ** = *p* < 0.01. Data for F2 at 100 nM was undertaken twice and not subjected to statistical analysis; (**C**) Binding of p53 to GST-E6. GST-E6-immobilised GS-beads were pre-incubated in the presence (lanes 2–6) or absence (lane 7) of aptamers F2 and F4, prior to addition of [^35^S]-p53 as described in panel A, except that data for F2 at 50 nM is not shown. GST-immobilised GS-beads were used as a control (lane 1). Binding (as a percentage of the no aptamer control) is shown graphically below the gel, however, the small amount of p53 pull down by GST was not quantified (lane 1). Data from a single experiment is shown. (**D**) Degradation of p53 by E6. [^35^S]-FLAG-E6 was pre-incubated in the presence (lanes 1–6) or absence (lane 7) of up to 400 nM F2 or F4, in buffer supplemented with rabbit reticulocyte lysate. A control reaction (lane 8) contained neither [^35^S]-FLAG-E6 nor aptamer. [^35^S]-p53 was added to all samples (lanes 1–8) for one hour at 29 °C. After incubation, samples were resolved on SDS-PAGE gels and analysed by autoradiography. The percentage of protein remaining (as a percentage of the no aptamer control) is shown graphically below the autoradiograph. Data from a single experiment is shown.

In order to to compare the effects of aptamers F2 and F4 on E6-PDZ interactions, pull-down assays were performed using two approaches. Firstly, the [^35^S]-labelled MBP-PDZ1 domain from Magi1 (synthesised in rabbit reticulocyte lysate) was allowed to bind to GST-E6 (expressed in *E. coli*) in the presence of up to 200 nM of F2 or F4. Binding was quantified by SDS PAGE and autoradiography of MBP-PDZ1 using a second Coomassie-stained gel as a control for GST-E6 loading. The data suggest that both F2 and F4 appeared to inhibit the E6-PDZ1 interaction in a dose-dependent manner, with F2 having the greater effect (Figure 4A). At 200 nM, F2 and F4 strongly inhibited the E6-PDZ interaction (by 84% ± 1.4% and 62% ± 9.5% respectively, comparing lane 9 with lanes 8 and 4). The reverse experiment was also performed, with [^35^S]-FLAG-E6 allowed to interact with recombinant MBP-PDZ-1 bound to amylose resin (Figure 4B). An E6Δ mutant (lacking the seven C-terminal amino acids) was included as a control. No pull-down of the PDZ1 domain was detected, as expected (Figure 4B, lane 7), although F2 inhibited the interaction in a dose-dependent manner as before (by 45.5% ± 3.6% at 200 nM; *p* < 0.01, Figure 4B comparing lanes 5 and 6) there seemed to be little effect of F4 even at 200 nM (Figure 4B, lane 2).

The ability of F2 to interfere with E6-PDZ1 binding, irrespective of the origin of the recombinant proteins, demonstrates the inhibitory activity of this aptamer and shows the utility of using bacterially-expressed proteins as SELEX targets. However, the data for F4 is less clear. It is possible that some of the apparent inhibition induced by F2 or F4 seen in Figure 4A could be due to E6 aggregation, rather than as a result of aptamer targeting. It is also possible that since bacterially-expressed E6 was used as bait for SELEX, this protein is recognised better by both aptamers than protein produced by cell-free translation (which is likely to include post-translational modifications, such as phosphorylation, which could affect aptamer binding).

The predicted secondary structures of F2 and F4 were calculated by Mfold. As the aptamer molecules used in this study have been modified against nuclease degradation by the inclusion of 2'F pyrimidines, the secondary structure predictions are unlikely to accurately reflect the structure of the aptamers. Such modifications in RNA are generally ignored in RNA folding studies and the presence of 2'F pyrimidines are likely to affect RNA conformation. However, it is interesting to note that a single structure was predicted for F2 (ΔG = −16.8 KJ·mol^−1^), but five for F4 (all with similar stabilities (ΔG= −15.8 to −16.6 KJ·mol^−1^), data not shown. The relative structural plasticity of F4 could explain why this molecule is a relatively poor initiator of apoptosis and why it is less effective at blocking E6-PDZ interactions. Further work (including mutation of both aptamer sequences) would be necessary order to address this and characterise the aptamers structurally.

### 2.4. Effect of F2 and F4 on E6-p53 Interactions

In order to probe the specificity of the effects of the E6 aptamers on the E6AP-independent binding of E6 to the C-terminus of p53, pull-down experiments were employed, utilising [^35^S]-p53 and GST-E6 in a similar way to that described above and as previously described [39]. A very small amount of pull-down of p53 was detected using GST alone (Figure 4C, lane 1), however, GST-E6 was able to efficiently pull-down p53 (Figure 4C, lane 7). No effects of F2 or F4 on this interaction were observed (Figure 4C, lanes 2–6) and it therefore appears that these molecules have no effect on the p53-E6 interaction.

In order to investigate the effect of aptamers on E6 degradation of p53 in the presence of E6AP, p53 degradation assays using rabbit reticulocyte lysate were performed [8]. p53 was readily degraded in the presence of E6, with only 8% of protein detectable after 1 h (Figure 4D, comparing lanes 7 and 8). However, the presence of F2 or F4 had little effect on p53 degradation (Figure 4D, lanes 1–6), even at very high aptamer concentration (up to 2 μM, data not shown). The ability of the aptamers (particularly F2) to target E6-PDZ interactions but not E6-p53 therefore suggests some specificity in their activity.

## 3. Experimental

### 3.1. Protein Expression and Purification

The HPV16 E6 coding sequence was cloned into the BamHI and EcoRI restriction sites of expression vector pGEX-2T containing an N-terminal GST tag. pETM-41/PDZI vector was used to express Magi1-PDZ1 (residues 448–560 of human Magi1) with a his-MBP tag at the N-terminus [40]. Expression of GST, GST-E6 and MBP-Magi1-PDZ1 proteins were performed in the BL21(DE3) strain of *E. coli* following induction at 30 °C with 0.5 mM IPTG for 3 h. To obtain GST and GST-E6 proteins immobilised on beads, bacterial cells collected from 50 mL of GST and GST-E6 expression cultures were lysed in 2.5 mL of Buffer A (PBS (140 mM NaCl, 2.7 mM KCI, 10 mM Na_2_ HPO_4_, 1.8 mM KH_2_PO_4_ pH 7.3), 2 mM DTT) in the presence of 1% Triton X-100, 0.5 mg/mL lysozyme, 5 µg/mL DNase I, 25 µg/mL RNase A, and anti-protease cocktail (EDTA-free, Roche, Welwyn Garden City, UK), cleared by centrifugation (30 min at 17500 × *g*, 4 °C) and filtration (0.22 µm, Millipore, Billerica, MA, USA) and incubated with 150 µL of glutathione-Sepharose 4B (GE Healthcare, Little Chalfont, UK) for 30 min at 4 °C. Protein-bound beads were washed six times with 30 volumes of Buffer A in the presence of 1% Triton X-100 and anti-protease cocktail, three times with Binding Buffer (50 mM Tris-HCl, pH 7.5, 100 mM NaCI, 2 mM DTT) and used immediately. To prepare MBP-Magi1-PDZ1 domain immobilised on beads, bacterial cells collected from 50 mL MBP-Magi1-PDZ1 of expression cultures were sonicated at 4 °C in 3 mL Buffer B (20 mM Tris-HCl, pH 7.5, 200 mM NaCI, 1 mM DTT, I mM EDTA) containing 5 mg/mL DNase I, 5 mg/mL RNase A and anti-protease cocktail, centrifuged at 17500 × *g* at 4 °C, filtered and incubated with 300 μL of amylose resin (New England Biolabs, Ipswich, MA, USA) for 1 h at 4 °C. Beads were washed six times with 30 volumes of Buffer B containing anti-protease cocktail, three times with 30 volumes of Binding Buffer and used immediately.

pcDNA3-p53(Arg) vector expressing wild-type p53 protein was a gift from Dr. Alan Storey (Weatherall Institute of Molecular Medicine, University of Oxford, Oxford, UK). Plasmids pSG5-E6 and pSG5-E6Δ were used for the expression of wild-type HPV16 E6 protein and E6 with deletion of the last seven amino acids, respectively. Both proteins carry an N-terminal FLAG tag. [^35^S]-labelled proteins (p53, FLAG-E6, FLAG-E6Δ and MBP-PDZ1 domain of Magi1) were synthesised using the TNT T7/SP6 coupled reticulocyte system (Promega, Madison, WI, USA) from pcDNA3-p53, pSG5-E6, pSG5-E6Δ and pETM-41/PDZI plasmids in presence of [^35^S]-methionine (Perkin Elmer, Waltham, MA, USA) according to the manufacturer’s protocol.

### 3.2. SELEX Procedure

The DNA template library used as the starting pool for selection was 5'-TGATAATACGACTCACTATAGGGAATGGATCCACATACTACGAAT-N_30_-TTCACTGCAGACTTGACGAAGCTT-3' (the T7 RNA polymerase recognition sequence is underlined). *In vitro* transcription reactions were carried out including 2'-fluoro-UTP and 2'-fluoro-CTP (TriLink Biotechnologies, San Diego, CA, USA) in a reaction containing 40 mM Tris-acetate (pH 8.0), 5 mM DTT, 1 mM EDTA, 10 mM magnesium acetate, 0.5 mM MnCl_2_, 8 mM spermidine, 2 mM each of ATP, GTP (Amersham Biosciences, Little Chalfont, UK), 2'-fluoro-UTP and 2'-fluoro-CTP (TriLink Biotechnologies), 1 U (per 50 µL reaction) of yeast inorganic pyrophosphatase (Sigma Aldrich, St. Louis, MO, USA) and 0.05 µL/µL of mutant T7 RNA polymerase Y639F (a gift from Peter Stockley, University of Leeds) and incubated for 3 h at 37 °C [41]. In each round of selection, RNA was incubated in Binding Buffer with GST-bound GS to remove molecules that bound to GST or the support matrix (negative selection) before incubation in Binding Buffer with GST-E6-bound GS. Bound species were isolated and amplified by reverse-transcription (Superscript II, Invitrogen, Life Technologies, Paisley, UK) and the cDNA amplified by PCR (KAPA 2 G Robust PCR Kit, Kapa Biosystems, Wilmington, MA, USA) to generate a new pool of template molecules. A total of 13 rounds of selection were performed. Because of the problems with E6 aggregation over time, it was not possible to perform selections robotically. Fresh samples of protein were used for each round. The resulting DNA was cloned into the pGEM-T Easy vector (Promega, Madison, WI, USA) and individual clones were sequenced and analysed. Sequence analysis was performed using the pGEMrp and pGEMup sequencing primers (5'-CCCAGTCACGACGTTGTAAAA CG-3' and 5'-CAGCTATGAACCATGATTACGCCAA-3', respectively). Sequence alignment was performed using the GeneBee multiple sequence alignment tool (AliBee). The sequence of the random region of the negative control aptamer SF1 was 5'-TCGGCTCAAAAATACGTCCGCACCATACA-3'. RNA was synthesised as previously described [33,36,42].

### 3.3. Cell Culture

SiHa [43] and C33A [44] cells (derived from human cervical carcinomas), and non-virally transformed HaCaT [45] cells were maintained in DMEM supplemented with 10% (v/v) FCS, 100 U/mL penicillin, 0.1 mg/mL streptomycin and 1% (w/v) glutamine in a humidified atmosphere at 37 °C and 5% (v/v) CO_2_.

### 3.4. Apoptosis Assays

Cells were transfected with up to 50 nM of aptamer RNA (as described in section 3.3 above) and maintained at 37 °C for 24 h. Cells were harvested by trypsinisation, washed twice with PBS and suspended in ice cold annexin V buffer (10 mM HEPES-KOH (pH 7.4), 140 mM NaCl and 2.5 mM CaCl_2_) with 5 μL FITC-conjugated annexin V (BD Biosciences, Franklin Lakes, NJ, USA) and incubated on ice for 15 min. Cells were co-stained with 5 μL propidium iodide (50 μg/mL) and analysed using the FACSCalibur and Cellquest Pro software (Becton Dickinson, Franklin Lakes, NJ, USA). Cells were treated with staurosporine at 500 nM for 24 h as a positive control for apoptosis.

### 3.5. Dephosphorylatin of Aptamer and qRT-PCR

De-phosphorylation of *in vitro* transcribed SF1 RNA was performed using 4 µM of aptamer, 10× reaction buffer, 20 U of RNase out and 10 U of Antarctic phosphatase (New England BioLabs, Ipswich, MA, USA) for 45 min at 37 °C. The reaction was inactivated at 70 °C for 10 min and phenol: chloroform extracted and ethanol precipitated. RNA was eluted in nuclease-free dH_2_O to obtain a 10 µM solution.

SiHa and C33A cells were transfected with 100 nM SF1 or dephosphorylated SF1 along with mock transfection and poly IC (1 µg/mL) as negative and positive controls. Cells were maintained at 37 °C for 24 h, prior to RNA extraction using Quick RNA Mini-prep kit (Zymo Research, Irvine, CA, USA) according to the manufacturer’s instructions. RNA was eluted in 35 µL of dH_2_O. cDNA synthesis were performed using a first-strand cDNA synthesis kit (Thermo Fisher Scientific, Basingstoke, UK), following manufacturer’s instructions. Approximately 1 µg of RNA (10 µL) was pre-incubated with 0.5 µg (1 µL) oligo (dT)_18_ for 5 min at 65 °C. Reaction buffer, 20 units U of RiboLock RNAase inhibitor, 2 mM dNTP mix, 20 U of M-MuLV Reverse Transcriptase were then added for 60 min at 42 °C. The enzyme was inactivated by incubation at 70 °C for 10 min. Quantitative real-time PCR (qPCR) was performed using the Quantifast SYBR Green PCR kit (Qiagen, Hilden, Germany) by a Corbett Rotor-Gene 6000 (Qiagen). cDNA (approximately 80 ng) 1 µM of both forward and reverse primers and 2× Quantifast SYBR Green PCR master mix were combined in a 25 µL total reaction. Primers utilised were MX1, IFNβ and U6, which were purchased from Qiagen. Conditions for PCR reactions were as follows: initial activation step and two-step cycle of denaturation, for 5 min at 95 °C and 10 s at 95 °C, respectively, followed by 40× repeats of combined annealing and extension steps for 30 s at 60 °C. A melting curve from 60 °C to 95 °C with 5 s at every 1 °C interval was performed at the end of last cycle. Data was analysed according to the ΔΔ Ct method described previously by Livak and Schmittgen, using the Rotor-Gene 6000 software [46].

### 3.6. Aptamer Binding Assays

Radiolabelled aptamer RNA was generated by 5' labelling with [γ^32^P]-ATP and unincorporated nucleotides were removed by column purification (NucAway spin columns, Ambion, Life Technologies, Paisley, UK). Labelled RNA (at a final concentration of 1–2 nM) was incubated with protein-loaded agarose-beads in binding buffer for 30 min at room temperature. The beads and any bound RNA were isolated from the reaction mixture and the supernatant transferred to scintillation fluid (unbound fraction). Beads were washed three times in binding buffer containing 50 µg/mL of BSA and suspended in scintillation fluid (bound fraction). The method is based on previously reported protocols [33,36,42].

Biotinylated HPV16 E6 N-terminal (MFQDPQERP) and C-terminal (RSSRTRRETQL) peptides (PeptideSynthetics, Fareham, UK) were immobilized on Dynabeads MyOne Streptavidin T1 according to the manufacturer’s protocol (Invitrogen/DYNAL, Life Technologies, Paisley, UK).

### 3.7. E6-PDZ Binding Assay

GST-E6-immobilised GS-beads (with 100 nM of GST-E6) were pre-incubated in binding buffer with aptamer RNA for 30 min at 4 °C prior to addition of [^35^S]-MBP-PDZ1 and incubation for 1 h. After incubation, beads were washed in binding buffer containing 50 µg/mL of BSA, and proteins resolved by SDS-PAGE. One gel was dried and analysed by autoradiography, a second gel was Coomassie-stained as a loading control. In parallel, [^35^S]-FLAG-E6 was preincubated with aptamer RNA at 4 °C. PDZ1-amylose resin (100 nM of PDZ1) was added and incubation continued for another hour. [^35^S]-FLAG-E6Δ was used as a control. After incubation the beads were washed and proteins resolved by SDS-PAGE. One gel was dried and analysed by autoradiography, a second gel was Coomassie-stained as a loading control.

### 3.8. E6-p53 Binding Assay

GST-E6-immobilised GS-beads (with 100 nM of GST-E6) were pre-incubated with aptamer RNA, prior to addition of [^35^S]-p53, as above. GST-immobilised GS-beads were used as a control.

### 3.9. p53 Degradation Assay

p53 degradation assays were performed as previously described [8] with the following modifications: 0.5 µL of [^35^S]-FLAG-E6 was pre-incubated in 15 µL of Binding Buffer for 30 min at 29 °C in the presence and absence of aptamer RNA. One µL of [^35^S]-p53 and 4 µL of rabbit reticulocyte lysate were added and incubation continued for another hour. Reactions were stopped with 20 µL of 2× SDS-loading buffer and analysed by 12% acrylamide SDS-PAGE followed by autoradiography.

### 3.10. Statistical Analysis

Standard errors are included and a student’s *t*-test was performed to obtain *p*-values, where appropriate.

## 4. Conclusions

We have described an RNA aptamer to HPV16 E6 that inhibits the interaction between E6 and the PDZ1 domain from Magi-1. This molecule (F2) is also the most apoptotic of the aptamers in SiHa cells, however, appears to have no effect on p53 degradation. It therefore appears that the apoptosis observed occurs via a p53-independent pathway, e.g., via BCL-2 family members, however, further work e.g., analysis of steady state levels of p53 in SiHa cells (in the presence and absence of aptamer) could be useful in order to confirm this finding. The ability of E6 aptamers to target E6-PDZ interactions highlights the benefits of such molecules as potential therapeutic or diagnostic agents in the future. However, it is unknown whether the aptamers are HPV16 specific and therefore studies are continuing to compare the effects of the aptamers on E6 from other HPV16 positive cell lines (e.g., CaSki) and with other HPV types and translate these (and the E7 aptamer [33,34]) studies into a system that better reflects human differentiated epithelium. We have previously demonstrated uptake of aptamer RNA by ketatinocytes, in the absence of transfection reagents [47]. It would also be interesting to exploit conjugation of aptamers to molecules that have been shown to facilitate internalisation into transformed cells [48].
